# Design of a High Density SNP Genotyping Assay in the Pig Using SNPs Identified and Characterized by Next Generation Sequencing Technology

**DOI:** 10.1371/journal.pone.0006524

**Published:** 2009-08-05

**Authors:** Antonio M. Ramos, Richard P. M. A. Crooijmans, Nabeel A. Affara, Andreia J. Amaral, Alan L. Archibald, Jonathan E. Beever, Christian Bendixen, Carol Churcher, Richard Clark, Patrick Dehais, Mark S. Hansen, Jakob Hedegaard, Zhi-Liang Hu, Hindrik H. Kerstens, Andy S. Law, Hendrik-Jan Megens, Denis Milan, Danny J. Nonneman, Gary A. Rohrer, Max F. Rothschild, Tim P. L. Smith, Robert D. Schnabel, Curt P. Van Tassell, Jeremy F. Taylor, Ralph T. Wiedmann, Lawrence B. Schook, Martien A. M. Groenen

**Affiliations:** 1 Wageningen University, Animal Breeding and Genomics Centre, Wageningen, The Netherlands; 2 Department of Pathology, University of Cambridge, Cambridge, United Kingdom; 3 Division of Genetics and Genomics, The Roslin Institute and the Royal (Dick) School of Veterinary Studies, University of Edinburgh, Roslin Midlothian, United Kingdom; 4 Institute for Genomic Biology, University of Illinois, Urbana, Illinois, United States of America; 5 Aarhus University, Faculty of Agricultural Sciences, Tjele, Denmark; 6 The Wellcome Trust Sanger Institute, The Wellcome Trust Genome Campus, Hinxton, Cambridge, United Kingdom; 7 INRA, Laboratoire de Génétique Cellulaire, Castanet Tolosan, France; 8 Illumina, Inc., San Diego, California, United States of America; 9 Department of Animal Science and Center for Integrated Animal Genomics, Iowa State University, Ames, Iowa, United States of America; 10 USDA, ARS, US Meat Animal Research Center, Clay Center, Nebraska, United States of America; 11 Division of Animal Sciences, University of Missouri-Columbia, Columbia, Missouri, United States of America; 12 Bovine Functional Genomics Laboratory, U.S. Department of Agriculture (USDA), Agricultural Research Service (ARS), Beltsville, Maryland, United States of America; Temasek Life Sciences Laboratory, Singapore

## Abstract

**Background:**

The dissection of complex traits of economic importance to the pig industry requires the availability of a significant number of genetic markers, such as single nucleotide polymorphisms (SNPs). This study was conducted to discover several hundreds of thousands of porcine SNPs using next generation sequencing technologies and use these SNPs, as well as others from different public sources, to design a high-density SNP genotyping assay.

**Methodology/Principal Findings:**

A total of 19 reduced representation libraries derived from four swine breeds (Duroc, Landrace, Large White, Pietrain) and a Wild Boar population and three restriction enzymes (*Alu*I, *Hae*III and *Msp*I) were sequenced using Illumina's Genome Analyzer (GA). The SNP discovery effort resulted in the *de novo* identification of over 372K SNPs. More than 549K SNPs were used to design the Illumina Porcine 60K+SNP iSelect Beadchip, now commercially available as the PorcineSNP60. A total of 64,232 SNPs were included on the Beadchip. Results from genotyping the 158 individuals used for sequencing showed a high overall SNP call rate (97.5%). Of the 62,621 loci that could be reliably scored, 58,994 were polymorphic yielding a SNP conversion success rate of 94%. The average minor allele frequency (MAF) for all scorable SNPs was 0.274.

**Conclusions/Significance:**

Overall, the results of this study indicate the utility of using next generation sequencing technologies to identify large numbers of reliable SNPs. In addition, the validation of the PorcineSNP60 Beadchip demonstrated that the assay is an excellent tool that will likely be used in a variety of future studies in pigs.

## Introduction

One of the main limitations to the dissection of economically important traits in livestock species has been the lack of a sufficient number of genetic markers for the development of high-density and high-throughput assays for association studies. The genetic regulation of quantitative traits is complex and the identification of the genes that underlie genetic variation requires large numbers of genetic markers, such as microsatellites or SNPs. To date, many QTL (quantitative trait loci) have been localized to large chromosomal regions in several species of domestic animals, including the pig [1], [2]. The need for more genetic markers is also supported by the extent of linkage disequilibrium (LD) in the pig genome, which has been estimated to extend from as little as 40–60 kb [3] up to 400 kb [4] in the commonly used commercial pig breeds, such as Duroc, Landrace and Large White. It has been predicted that a marker density of 5–10 markers per cM (centiMorgan) will be needed to conduct whole genome association studies in the pig [4], [5]. However, until recently the identification of such markers *en masse* has been a very challenging and costly task.

In recent years, new sequencing technologies have emerged which offer great promise for marker discovery due to their ability to efficiently generate large amounts of sequence data, both in terms of time and cost. They are usually referred to as “second generation” or “next generation” sequencing technologies and include the Illumina Genome Analyzer (previously Solexa), Roche's 454 FLX system and Applied Biosystems' SOLiD. The chemistry used by each technology, as well as the read length and the sequence output vary [6]. To date, these instruments have been widely used for genome sequencing and re-sequencing and SNP discovery [6].

Genome assemblies are being produced for a growing number of animal and plant species, including several species of domestic animals like the cow [7], chicken [8], dog [9], cat [10] and pig [11], [12]. The availability of the genome assemblies for the relevant species and the ability to acquire massive sequence data from next generation sequencers allows the efficient identification of a large number of genetic markers, in particular SNPs [13] and small indels (insertions/deletions).

The most efficient way to genotype large numbers of SNPs is to design a high-density assay that includes tens of thousands of SNPs distributed throughout the genome. These SNP “chips” are a valuable resource for genetic studies in livestock species, such as genomic selection [14], detection of QTL or diversity studies. Recently, high-density SNP chips have become available for cattle [15], horse and dog (http://www.illumina.com; http://www.affymetrix.com), including the first application of a dog SNP assay [16], but no such tool existed for pigs. Hence, the objectives of this study were to: 1) perform a large-scale, genome-wide porcine SNP discovery study using next generation sequencing; 2) develop and characterize a high-density porcine SNP chip using *de novo* discovered and other SNPs from public sources.

## Materials and Methods

### Animals and DNA samples

DNA samples were obtained from five pig breeds, including Duroc (DU), Pietrain (PI), Landrace (LR), Large White (LW) and Wild Boar (WB). For each breed, a single DNA pool containing equal DNA amounts from all of the samples was prepared. The number of animals per pool was 34, 23, 29, 36 and 36 for DU, PI, LR, LW and WB, respectively. The DNA samples for the commercial breeds were representative of the worldwide distribution of the breeds (samples originated from the USA, Netherlands and Denmark), whereas the WB samples were collected mainly in Europe, with five samples originating from Japan.

### Construction of the reduced representation libraries

For each breed, a total of 25 µg from each DNA pool, divided in five aliquots of 5 µg each, was digested with each of three restriction enzymes *Alu*I, *Hae*III and *Msp*I (Fermentas GmbH, St. Leon-Rot, Germany), following the manufacturer's recommendations. For each restriction digest, 10 units of enzyme were used in a final volume of 10 µl and incubated at 37°C for 16 hr. Upon completion of each digestion, fragments were fractionated with a 10% non-denaturing polyacrylamide Criterion TBE gel (Biorad, Veenendaal, Netherlands) at 100 V for 190 min. DNA fractions were stained by immersing a gel for 15 min in a TBE 1× solution containing ethidium bromide. Fragments in particular size ranges were excised from the gel. The size ranges for fragments excised differed between libraries and included fragments of 160–200 bp (*Alu*I short), 200–240 bp (*Alu*I long), 160–200 bp (*Hae*III) and 100–200 bp (*Msp*I). After excision, the gel pieces were placed in a 0.5 ml microcentrifuge tube that had been pierced in the bottom, and then placed in a collection tube and centrifuged for seven min at 16000×g. A total of 400 µl of recovery buffer was added to the disrupted gel pieces, and the samples were kept overnight at 4°C. DNA was purified from the gel slurry by passing the samples through Sample Filter Cups from the DNA Gel Extraction Kit (Millipore, Billerica, MA, USA) by centrifugation at 4965×g for 10 min at room temperature. Next, 200 µl of recovery buffer was added and the samples were centrifuged at 16000×g for 15 min, followed by an ethanol precipitation. DNA was dissolved in 40 µl of Puregene DNA Hydration Solution (Gentra Systems, Valencia, CA, USA). Samples were quantified by spectrophotometry, and at least 300 ng of purified DNA was used to construct a RRL (reduced representation library) for sequencing. A total of 19 breed specific RRL were prepared, including 4 libraries each for DU, PI, LR and LW, but only three libraries were available for WB, because the *Alu*I short library was not prepared.

### Sequencing

All 19 libraries were sequenced on a 1G Genome Analyzer (Illumina, San Diego, CA, USA). The length of all sequences generated in this study was 36 nucleotides. In addition, two pooled samples derived from each of the *Alu*I RRLs were prepared and sequenced using a 454 FLX system (Roche Applied Science, Indianapolis, IN, USA) on the GS FLX platform. For the Genome Analyzer, library constructions followed the protocol supplied with the Genomic DNA Sample Prep Kit (Illumina, “Preparing Samples for Sequencing Genomic DNA”, version 1003806_Rev. B, March 2008) with minor modifications. Library construction started with the addition of an adenosine base to the 3′ end of the DNA fragments, except for the *Msp*I digested samples which were initially end repaired. The volumes of adapter reagent used in the adapter ligation reactions were titrated accordingly to the amount of input DNA to maintain a 10∶1 molar ratio of adapter to DNA. The concentrations of the libraries were determined using a NanoDrop ND-1000 Spectrophotometer (Saveen and Werner AB, Limhamn, Sweden) and the size and purity were determined using an Agilent 2100 Bioanalyzer in combination with the Agilent DNA 1000 Kit (Agilent Technologies, Nærum, Denmark). The libraries were diluted in buffer EB (QIAGEN) to 10 nM, denaturated with 2 N NaOH to a final DNA concentration of 0.5 nM, diluted to 2–4 pM with pre-chilled Hybridization buffer (Illumina) and loaded in individual lanes into a 1.0 mm flowcell together with a single lane of a 2 pM PhiX control library (Illumina). Following cluster amplification, linearization, blocking, and primer hybridization, 36 cycle sequencing were conducted on an Illumina Genome Analyzer (version I) using the Genomic DNA Sequencing Primer in combination with clustering and sequencing kits supplied by Illumina. The resulting images were transferred to the pipeline computer and analyzed using the Genome Analyzer Pipeline Software (version 1.0, Illumina) generating the raw fastq files. For FLX sequencing, the blunt fragments produced by *Alu*I digestion were ligated directly to the adapters provided in the library preparation kit without the shearing, sizing or end-polishing steps. Single stranded libraries were then prepared and sequenced according to the manufacturer's instructions. The *Alu*I short and *Alu*I long libraries were sequenced using 5.5 and 4.5 full machine runs, respectively, using the two-region gasket.

### Processing of the GA sequences

The raw sequence data were filtered according to different criteria. Each sequence was first evaluated for the presence of the expected sequence motif for each restriction enzyme; sequences not containing the expected sequence were discarded. The acceptable sequences began with CT, CC or CGG for the *Alu*I, *Msp*I, and *Hae*III restriction digests, respectively. Sequences that contained the same nucleotide at more than 18 continuous positions were also eliminated. The average quality score was next calculated for each read by averaging the individual score for each of the 36 base positions. Previous results obtained in a smaller pilot study [17] indicated that a minimum average quality score of 12 was an acceptable threshold, and, therefore, all sequence reads with an average quality score<12 were removed from the dataset. Finally, sequences were also filtered for the presence of over-represented reads. For this purpose, we calculated the sequence depth for each RRL as the average of the total number of reads that aligned to each unique position in the reference genome. Reads that were present more than 5× the estimated sequence depth were removed from the dataset.

### Reference genome

The reference genome against which the 36 bp sequences were aligned included all the porcine autosomes and the X chromosome. The state of completion of the assembly of each chromosome varied because the sequencing of the pig genome has not yet been completed. This genome sequencing and assembly effort is being performed under the direction of the international Swine Genome Sequencing Consortium [11] using a hierarchical shotgun sequencing strategy in which BAC clones from a minimum tiling path of clones are selected from the highly contiguous physical map of the pig genome [18]. These BAC clones are being sequenced to a minimum of 4-fold sequence coverage. The tiling path was selected from clones from the CHORI-242 BAC library generated from a single Duroc sow. Sequence data have been assembled into contigs on a BAC-by-BAC basis and have immediately been released into the EMBL/Genbank/DDBJ DNA sequence databases. BAC contig sequences have been assembled on a genome-wide basis at regular intervals. The unmasked “PreEnsembl” version of the *Sus scrofa* assembly 7 of the pig genome available on July 28^th^ 2008 was used for this study, and that resource was obtained from: ftp://ftp.sanger.ac.uk/pub/S_scrofa/assemblies/PreEnsembl_Sscrofa7/.

To represent regions of the genome that had not yet been assembled, two pseudo chromosomes were built from 454 sequence data generated from the two *Alu*I libraries. For each library, all sequences were first aligned to the *Sus scrofa* assembly with BLAT [19]. Sequences that did not significantly align to that assembly were next used to create consensus sequences (contigs). Our strategy was to employ a simpler version of the approach used by TIGR to assemble large shotgun sequencing projects [20]. A Perl script was developed to cluster sequences which had the same motif in the first 32 bp. Batches of clusters were then assembled using CAP3 [21], producing a set composed of contigs and singletons (unique sequences) which were used to build a pseudo chromosome in which sequences were separated by blocks of 100 Ns.

### SNP discovery

Sequences were aligned against the assembled reference genome and initial SNP detection was performed using MAQ [22]. For SNP discovery, only reads that aligned to a single unique location of the genome were considered. Because MAQ calls a SNP as being any difference between the reads or between the reads and the reference genome, the initial MAQ SNP prediction output is large and must be filtered. Several criteria were used to exclude the less reliable SNPs from the dataset. Thresholds were established for a number of MAQ values that were useful in predicting reliable SNPs. Specifically, MAQ's minimal map quality for the read, minimal consensus quality and minimal map quality of the best mapping read for each predicted SNP position were used as criteria to select reliable SNPs by setting the thresholds for all three parameters at 10 (SNPs with any values<10 were discarded). Moreover, we required the minor allele at each SNP be represented in at least three reads and that the total number of reads per SNP was lower than 120 (SNPs with higher read depth were discarded). The final SNP set comprised the *de novo* identified SNPs that had passed all filters. Finally, SNP MAFs were estimated by directly counting the number of reads for each allele. All *de novo* identified SNPs have been submitted to dbSNP (accession numbers from ss131027063 to ss131629651). The flanking sequences for the SNPs used to build the genotyping assays were derived from the ongoing sequencing of the porcine genome by the Sanger Institute and these sequences are regularly uploaded to Genbank.

In addition to these *de novo* identified SNPs, the final porcine SNP dataset also comprised previously identified SNPs that were available from different public sources, including SNPs detected in a pilot study conducted at Wageningen University using a *Dra*I RRL [17], SNPs derived from the 7K porcine Illumina Custom Infinium Bead SNP Chip, SNPs identified by high throughput pyrosequencing [23], SNPs discovered through Sanger sequencing at INRA and publicly available SNPs from dbSNP, including a collection of porcine SNPs, derived from several sources, collected by the University of Cambridge.

### Initial SNP validation

Information on 44,236 SNPs derived from the *Hae*III library with flanking sequence information was available for validation of the SNP calling procedure. These markers were sorted by predicted location within the genome, and every fortieth sequence was processed using the Sequenom Assay Design software. From the output, assay groups were designed to have 32 multiplexed SNPs per well. Critical features to be tested were the position of the SNP within the GA sequence read, average quality score of the most frequently called base, number of alleles identified, as well as position within the genome. Nine assay groups (288 individual SNPs) were selected to represent SNPs in all tested categories. Reactions were set up per manufacturer's instructions for the Sequenom iPLEX GOLD chemistry and the 128 boars that contributed to the commercial breeds RRLs were individually genotyped to allow the determination of the true SNP allele frequencies in the discovery population.

### Selection of the final SNP list

A list which included the final porcine SNP dataset comprising 549,282 *de novo* identified and publicly available SNPs, was submitted to Illumina for design score calculation, which was performed with Illumina's Assay Design Tool for Infinium. Type II Infinium SNPs were prioritized for selection in the design of the assay because they require only one bead type on the chip. In addition to the Illumina design score and the type of Infinium assay, we also considered other parameters, such as the estimated SNP MAF, spacing of the SNPs along each chromosome, genome-wide coverage (number of SNPs selected for each chromosome), presence of other SNPs within 40 bp of each target SNP and available information concerning the conversion rate of the SNP (i.e., had the SNP been assayed successfully before?). Finally, any SNP known to be linked to a patent was excluded from the list. Information for these parameters was collected for all available SNPs and was used to assign SNPs to waves [15]. A total of 22 waves were formed and used to conduct rounds of SNP selection. The first wave contained only type II Infinium SNPs with a design score≥0.8 and MAF≥0.25. In the second wave the MAF threshold was lowered to 0.15, while in the third wave the design score threshold was lowered to 0.7 while the MAF was again set to 0.25. As far as possible, the SNPs included on the Beadchip were selected from the waves with high stringency followed by additional rounds of selection in which the selection criteria were gradually relaxed. Upon completion of the selection process, the SNP list was manually inspected for the presence of large gaps (>250 kb) between SNPs. The identified large gaps were visually inspected and the size of the gaps decreased by manually selecting SNPs to reduce the size of the gap, whenever possible.

Because the available porcine genome sequence (build 7) only covered 70% of the genome, 70% of the SNPs selected to be included on the assay came from those for which position on genome build 7 was known. To ensure a complete coverage of the porcine genome, the remaining 30% of SNPs were selected from the list of SNPs that could not be mapped to porcine genome build 7.

### Validation of the Illumina Porcine 60K+SNP iSelect Beadchip

To evaluate the performance of the Illumina iSelect BeadChip, which is now commercially available as the PorcineSNP60, the panel of 64,232 markers that passed the assay design tool informatics screen was used to genotype all 158 individuals from the five breeds that had been included in the original discovery panel. Samples were genotyped in the Illumina services lab and data was evaluated for robust genotyping [24]. Markers were evaluated for signal intensity, robust cluster formation and cluster separation. The DNA sample set included 6 replicate pairs and 94 trios to aid in cluster evaluation. The 62,121 loci that passed validation comprise the PorcineSNP60 panel.

### Correlation between MAFs derived from sequencing and genotyping

Since allele frequency data were available from both the sequencing and genotyping efforts we investigated the relationship between the allele frequency estimates derived from both approaches. To obtain the best possible estimate for the correlation we applied some filters to the dataset. We used only SNPs that had been identified in the four RRLs prepared for this study (*Alu*I short, *Alu*I long, *Hae*III and *Msp*I) and that had not been mapped to the sex chromosomes. Since the X chromosome contains pseudoautosomal regions that are largely unknown it would not be possible to correct for the effect of gender. For each SNP the reference allele was set as the major allele over all breeds as derived from sequencing. For each of the breeds the sequence derived frequency was matched with the genotype derived frequency. The genotyping was performed on the same individuals that were used to make the sequencing pools. Comparisons between sequence-derived and genotype-derived frequencies were done on a breed by breed basis since the number of alleles counted per breed could vary widely between breeds. Correlations were calculated using Pearson's product-moment correlation as implemented in R (www.r-project.org). The correlation between genotype-derived and sequence-derived frequencies was plotted against sequence depth and fitted using locally weighted scatter plot smoothing (LOESS function in R).

## Results

### Sequencing of the reduced representation libraries

A total of seven restriction enzymes were tested for the construction of the RRLs, including *AluI, HaeIII, MspI, PvuII, RsaI, ScaI* and *StuI*. Restriction enzymes producing blunt end fragments were chosen because this would result in DNA fragments that could be directly ligated to the adaptors used in the preparation of libraries for sequencing on the Illumina Genome Analyzer. We selected *Alu*I, *Hae*III and *Msp*I because these enzymes maximized the number of fragments detected in the desired size range and minimized the presence of repetitive elements [13].

The total number of generated sequences surpassed 370 million and after filtering based on the quality criteria described in the materials and methods, over 247 million reads remained for SNP discovery using MAQ (Table 1). The estimated genome coverage for the different RRLs varied from 1.5 to 3.5% and the combined 4 RRLs span about 10% of the porcine genome.

**Table 1:** Number of Illumina Genome Analyzer reads generated, filters applied to the dataset and final number of reads used for SNP discovery from the four RRLs.

	*Alu*I short	*Alu*I long	*Hae*III	*Msp*I	Total
**Starting number of reads**	87,962,916	145,926,417	67,057,081	69,507,210	370,453,624
**Filters**	**Number of reads removed from dataset**				
**Restriction enzyme motif**	3,276,584	48,401,718	6,854,265	16,555,378	75,087,945
**Poly-(A,C,G,T)**	260,648	656,411	278,435	202,585	1,398,079
**Quality score**	1,004,585	1,085,621	2,138,675	402,036	4,630,917
**Over-represented reads**	10,167,678	14,193,925	9,559,415	7,622,490	41,543,508
**Number of reads used for mapping**	73,253,421	81,588,742	48,226,291	44,724,721	247,793,175
**% Usable reads**	83.3	55.9	71.9	64.4	66.9

To identify SNPs in the 30% of the porcine genome that was not represented in genome build 7, the two largest RRLs (*AluI* short and *AluI* long) were also sequenced on Roche's 454 platform. The total number of sequences generated on this platform was 2,121,435 and 1,922,150 for the *AluI* short and *AluI* long RRLs, respectively.

### Reference genome

The genome of the pig is comprised of 18 autosomes and the X and Y sex chromosomes with an estimated total size of 2.7 Gb similar to that of human. The reference genome (build 7) against which the GA sequences were aligned for SNP discovery, represented approximately 70% of the porcine genome with the state of sequence completion of each chromosome differing from less then 50% to up to 99% for individual chromosomes. To discover SNPs in the 30% of the porcine genome not represented in the assembly, the 454 sequences from the two *AluI* libraries were used. All 454 sequences were first aligned against sequences from the genome build 7 and only unique 454 sequences not represented in build 7 were used. These remaining 454 sequences were used to generate unique sequence contigs, which were then concatenated into two pseudo chromosomes (*Alu*Ishort and *Alu*Ilong, respectively). The final extended reference genome used with MAQ and to which all GA sequences were aligned consisted of porcine genome build 7 and the two pseudo 454-*Alu*I chromosomes. Build 8 of the pig genome, released in October 2008, was also used to compare the distances between SNPs calculated using builds 7 and 8 of the pig genome.

### SNP discovery

All 247 million sequences that passed our selection criteria (Table 1) were aligned to the extended reference genome using MAQ. MAQ aligns all GA sequences to the reference genome and reports all variation detected within the GA sequences as well as any difference between the GA and the reference sequences. The initial raw output from MAQ consisted of over 9 million positions at which variation was detected. The majority of these do not represent SNPs but result from sequencing errors in the GA sequences, errors in the reference genome or the alignment of paralogous sequences. However, after applying the filters described in the materials and methods, 315,130 variations remained representing high confidence SNPs (Table 2). While only SNPs for which the minor allele was represented by at least 3 sequences were considered in the selection of SNPs for the Beadchip design, we also identified 57,756 SNPs for which the minor allele was represented by only two sequences (low MAF SNPs) (Table 2). Initial validation of putative SNPs from the *HaeIII* RRL showed that a good indicator of the likelihood of SNP failure was the quality of the nucleotide at the SNP (low quality bases were more often associated with non-converting or monomorphic SNPs). Hence, we examined the quality score at each of the SNP bases in the two reads and established a threshold of 20 to discover reliable SNPs in those cases where the minor allele was seen only twice. The final total number of high confidence SNPs identified by using these strategies was 372,886.

**Table 2:** Summary of the SNP discovered from the four analyzed RRLs.

	*Alu*I short	*Alu*I long	*Hae*III	*Msp*I	Total
**Initial MAQ output**	2,625,323	2,854,329	2,377,571	1,180,640	9,037,863
**Filtered SNP output**	106,456	124,578	56,817	27,279	315,130
**Low MAF SNPs** 1	11,149	39,096	5,620	1,891	57,756
**Total High confidence SNPs**	117,605	163,674	62,437	29,170	372,886

1 SNP detected with only two minor alleles among the sequence reads.

**Figure 1:**
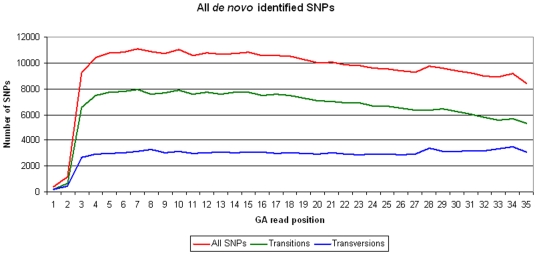
SNP distribution on each of the GA read positions. The distribution represents all *de novo* identified SNPs, from the RRLs generated. The number of transitions and transversions identified is also illustrated.

**Figure 2:**
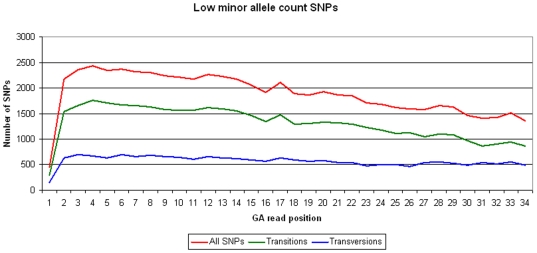
Distribution of the low minor allele count SNPs on each of the GA read positions. For these SNPs two reads were identified for the minor allele. The number of transitions and transversions identified is also illustrated.

To further evaluate the conversion rate of the SNPs discovered by our approach we looked at the ratio between the number of transitions and transversions. For those SNPs where the minor allele was seen at least three times, 69.4% were transitions and 30.6% were transversions. Almost the same values were found for the 57,756 additional low MAF SNPs identified using the alternative thresholds (69.6% transitions and 30.4% transversions). Finally, because the error rate in GA sequences increases towards the end of the sequences, we also plotted the distribution of the identified SNPs against the nucleotide position within the GA sequences (Figures 1, 2).

### Initial SNP validation

Before selecting the final 64,232 SNPs to be included on the Beadchip, we decided to validate the selection criteria used during the SNP discovery phase by genotyping a representative sample of 288 SNPs in the individuals used to create the discovery panel. Of the 288 assays attempted on the Sequenom MassArray platform, 241 (83.7%) assays yielded a sufficient number of genotypes to draw useful conclusions from the data (>70% call rate) across all 128 DNA samples. Of the 241 assays, 20 were monomorphic in the DNAs of the boars used to generate the RRL libraries (8.3%). An assessment of the quality score of the predominant base indicated that when the most predominant base scored was of low quality the frequency of monomorphic assays was tenfold higher than when the predominant base called was of high quality (44.0% vs 4.2%). We, therefore, added this as an additional selection criterion for SNP discovery. Twenty-four successful Sequenom assays were designed for SNPs with 3 alleles detected in sequencing the RRL. Only one of the 24 assays was actually tri-allelic, while two of the assays were monomorphic, representing a total drop-out rate of 12.5%. The average read depth in the RRL for the 288 SNPs tested was 37.4. Assays designed for SNPs with over 2× of the mean read depth resulted in 23.5% monomorphic assays (4 of 17) and 11.8% of assays with excess heterozygotes (2 of 17), likely due to duplicated regions in the genome. While an initial assessment of position within the read indicated that as the SNPs were located towards the end of a read the percentage of monomorphic markers increased, removal of SNPs detected with low quality bases eliminated this trend.

### Design of the 60K SNP Beadchip

Besides the *de novo* generated SNPs, an effort was made to assemble a collection of porcine SNPs from other public sources. The final combined SNP collection surpassed 549K SNPs and this set was used to select the SNPs that were included on the PorcineSNP60 Beadchip. The information regarding the results for the Beadchip design is summarized in Table 3. The total number of SNPs submitted to Illumina for assay design was 72,000 and a total of 64,232 SNPs passed decoding and were included on the Beadchip. The information regarding the SNP sources used, as well as the number of SNPs available for selection and included on the Beadchip for each source is summarized in Table S1. Of the total number of selected SNPs, 45,510 were mapped to a specific chromosome in porcine genome build 7, including 21 SNPs on chromosome Y, while 18,722 were unmapped (Table 3). However, we were able to predict the position of 4,193 of these SNPs by comparative mapping. A total of five mitochondrial SNPs were also included.

**Table 3:** Summary of SNPs included on the Beadchip, assay conversion, Infinium SNP type, wave number and estimated distances between SNPs using builds 7 and 8 of the pig genome (total numbers and breakdown by chromosome).

SSC	SNPs on Beadchip	Assay		Infinium type		Wave number				Distance between SNPs (kb) build 7			Distance between SNPs (kb) build 8		
		Working	Not working	I	II	1	2	3	≥4	Average	Largest	Gaps≥250 kb	Average	Largest	Gaps≥250 kb
1	6,584	6,477	107	138	6,446	88	4,705	512	1,279	37.7	461.7	14	39.0	545.3	19
2	2,441	2,381	60	33	2,408	69	2,038	150	184	32.4	222.3	0	36.9	409.3	13
3	1,907	1,856	51	15	1,892	54	1,577	128	148	33.7	224.1	0	38.4	445.8	11
4	3,690	3,631	59	60	3,630	107	2,798	259	526	34.7	355.4	6	34.8	355.3	11
5	2,186	2,137	49	16	2,170	49	1,705	156	276	34.6	272.8	2	37.1	367.5	10
6	1,411	1,375	36	5	1,406	32	1,214	67	98	32.2	162.1	0	37.1	375.0	6
7	3,669	3,567	102	70	3,599	70	2,764	235	600	36.3	438.4	12	36.4	598.2	14
8	1,924	1,888	36	13	1,911	32	1,597	149	146	34.8	331.3	3	40.1	618.8	16
9	2,390	2,348	42	26	2,364	42	2,042	159	147	33.4	281.4	2	37.7	519.9	13
10	1,270	1,221	49	6	1,264	18	1,167	39	46	32.7	169.9	0	35.2	377.9	3
11	1,827	1,786	41	10	1,817	16	1,533	122	156	34.9	281.6	1	36.1	360.7	4
12	932	913	19	3	929	34	834	26	38	31.3	169.0	0	33.7	294.6	2
13	3,297	3,229	68	24	3,273	69	2,649	255	324	36.2	449.6	3	38.1	449.5	5
14	4,153	4,062	91	73	4,080	95	3,185	238	635	35.8	371.8	36	35.8	371.8	0
15	2,426	2,370	56	53	2,373	34	1,853	184	355	39.6	449.6	8	42.8	579.6	20
16	1,458	1,429	29	14	1,444	16	1,290	71	81	35.0	198.2	0	37.2	327.0	1
17	1,659	1,622	37	11	1,648	26	1,370	104	159	33.6	259.0	1	34.2	295.3	2
18	1,037	1,020	17	5	1,032	37	802	98	100	34.1	161.2	0	37.6	306.6	3
X	1,228	1,168	60	65	1,163	9	740	115	364	59.2	447.8	27	67.5	955.5	54
Y	21	19	2	0	21	0	0	0	21	-	-	-	-	-	-
Unmapped	14,529	14,050	479	118	14,411	132	14,100	135	162	-	-	-	-	-	-
Unmapped (predicted position)	4,193	4,072	121	74	4,119	259	3,045	448	441	-	-	-	-	-	-
**TOTAL**	64,232	62,621	1,611	832	63,400	1,288	53,008	3,650	6,286	-	-	115	-	-	207

**Figure 3:**
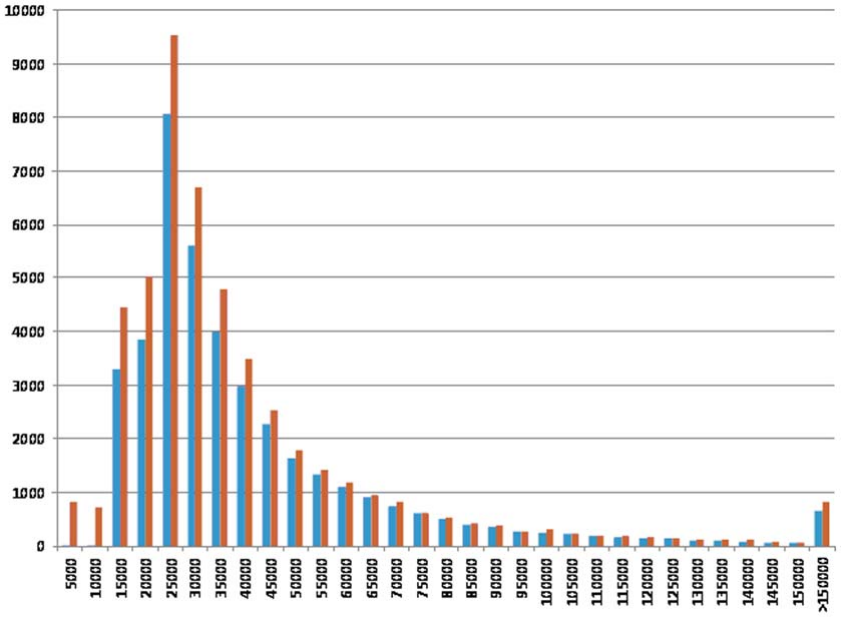
Distances between the SNPs included on the 60K+porcine Beadchip. The distances (x axis) were calculated using builds 7 (blue) and 8 (red) of the pig genome sequence assembly.

The selection of SNPs to be included on the Beadchip was based on specific wave numbers assigned to each individual SNP based on quality parameters, MAF, type of assay (Infinium type I or II) and location in the genome. Final SNP selection was performed in waves [15] resulting in only 832 or 1.3% of the total Infinium I SNPs being selected. Over 90% of the SNPs included on the Beadchip were selected in the first three waves, where SNP quality was the highest. With the exception of the X chromosome, the average distance between SNPs on build 7 is between 30 and 40 kb (Figure 3). Nevertheless, larger distances between SNPs were found on all chromosomes, with the largest distances being detected on chromosomes 15 (407.4 kb) and X (447.8 kb). There were 115 intervals with a gap size larger than 250 kb. Most of these large gaps are located on chromosomes 14 and X, where 36 (SSC14) and 27 (SSCX) large gaps were detected However, there were also chromosomes with no large gaps between the SNPs (chromosomes 2, 3, 6, 10, 12, 16 and 18). For most of these regions with large gaps there were simply no SNPs available for selection.

After the initial SNP discovery and our design of the Beadchip a new build of the pig genome became available (build 8). To increase the number of SNPs mapped to a specific position within the genome, the flanking sequences for all 64,232 SNPs were aligned to the new build using BLAT [19]. This allowed us to position an additional 4,215 previously unmapped SNPs onto the porcine genome and allowed us to verify the accuracy of our predictions based on the comparative mapping approach. Of the 4,193 SNPs with a predicted map location, 1,030 specifically aligned to porcine genome build 8. Only 68 of these SNPs mapped to a location that differed from our prediction, with the majority of the SNPs (93%) mapping in accordance to the predicted location. There were 42 SNPs with conflicting map positions on genome builds 7 and 8, and 411 SNPs positioned on genome build 7 that did not align to build 8. The number of large gaps between adjacent SNPs increased when build 8 was used, with 207 gaps>250 Kb identified using this build. This increase was observed for all chromosomes except for SSC14, where the number of large gaps decreased to zero, indicating that there were unmapped SNPs from build 7 that mapped to SSC14 in build 8, filling the observed large gaps.

### Validation of the 60K SNP Beadchip

The panel of 64,232 porcine SNPs was used to genotype the 158 individuals from the five pools that were used for SNP discovery. SNPs were validated for robust genotyping quality by evaluating signal intensity and cluster separation. From the initial panel of SNPs, 62,121 (97.5%) genotyped successfully, while 1,611 SNPs (2.5%) could not be reliably genotyped. Of the 62,621 validated loci, 58,994 were polymorphic which indicates that the SNP conversion success rate was 94%. Furthermore, for 57,109 SNPs, the MAF was>5% and for 58,737 SNPs the MAF was>1%. The average MAF for all scorable SNPs was 0.274 (Table 4). The DNA sample set used for assay validation included 6 replicate pairs and 94 trios. The reproducibility rate was 100% for 350,312 genotype comparisons, and the Mendelian consistency rate was 100% for 5,478,678 genotype comparisons.

**Table 4:** Description, by SNP source, of the number of working SNPs, SNPs by minor allele frequency and monomorphic SNPs.

SNP source^1^	SNPs	Working SNPs	Non working SNPs	MAF>0.05	MAF>0.01	MAF = 0
ALGA	20,144	19,593	551	18,236	18,436	1,133
ASGA	15,310	14,994	316	14,530	14,687	299
BGIS	117	115	2	34	43	71
CADI	21	20	1	12	13	7
CAHM	32	26	6	6	7	3
CAIL	17	15	2	6	7	8
CAMB	9	8	1	0	0	7
CAPE	6	5	1	1	1	4
CASI	550	542	8	444	493	37
DBKK	20	19	1	10	14	5
DBMA	21	21	0	20	20	1
DBNP	45	45	0	24	34	11
DBUN	39	37	2	15	21	13
DBWU	96	94	2	90	93	1
DIAS	1,202	1,171	31	1,144	1,155	15
DRGA	3,422	3,347	75	2,912	3,151	177
H3GA	6,300	6,135	165	5,740	5,809	321
INRA	2,528	2,493	35	1,802	2,174	244
ISU	37	37	0	34	36	1
M1GA	1,828	1,779	49	1,719	1,748	29
MARC	12,121	11,760	361	9,976	10,438	1,235
SIRI	324	323	1	313	316	4
UMB	35	35	0	35	35	0
WUR	8	7	1	6	6	1
**TOTAL**	64,232	62,621	1,611	57,109	58,737	3,627

1 The description for these acronyms is summarized in Table S1.

**Figure 4:**
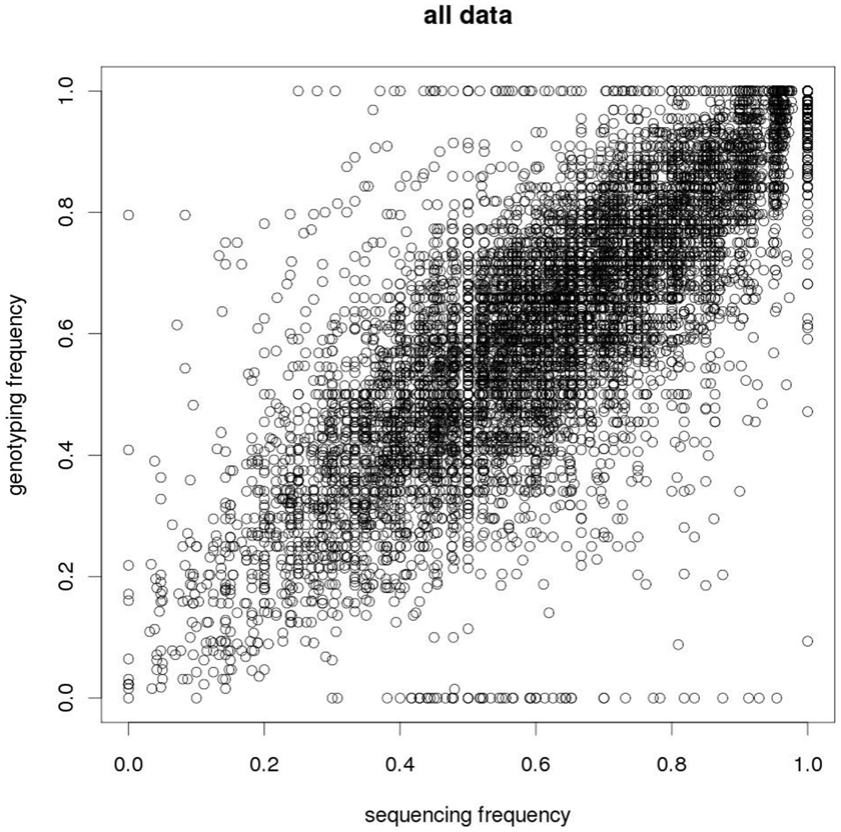
Correlation between sequence-derived and genotype-derived allele frequencies. The scatter plot was determined using the frequencies of the PorcineSNP60 SNPs derived from the RRLs generated.

**Figure 5:**
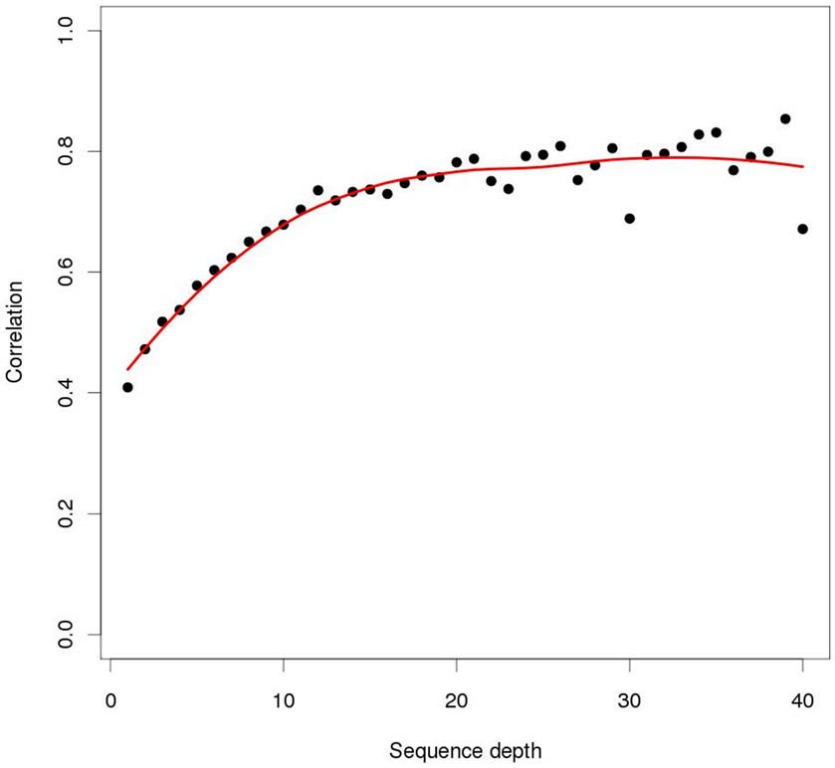
Relationship between sequence depth and the correlation between sequence-derived and genotype-derived allele frequencies. This relationship was determined for the PorcineSNP60 SNPs derived from the RRLs generated.

Correlations between sequence-derived and genotype-derived allele frequencies were approximately 0.8 at sequencing depths of 20 or higher (Figure 4, Figure S1). Even at lower sequencing depths, sequence-derived allele frequencies were highly predictive of the actual frequencies, as estimated from genotyping the individuals in the pools (Figure 5). At sequence depths greater than 20, little increase in correlation was observed. Some variation exists in correlations between libraries (e.g., the *Msp*I RRL presented higher correlation values when compared with the other RRLs), although these differences were small (Table 5).

**Table 5:** Values for the correlation between sequence-derived and genotype-derived allele frequencies for different read depths and SNP sources.

Read Depth	SNP source									
	All SNPs	Duroc	Landrace	Large White	Pietrain	Wild Boar	*Alu*I short	*Alu*I long	*Hae*III	*Msp*I
**>0**	0.53	0.61	0.49	0.49	0.52	0.49	0.59	0.48	0.59	0.61
**>5**	0.68	0.73	0.69	0.64	0.66	0.69	0.67	0.66	0.71	0.76
**>9**	0.74	0.77	0.75	0.70	0.72	0.76	0.73	0.72	0.75	0.81
**>19**	0.79	0.80	0.82	0.74	0.77	0.78	0.79	0.76	0.79	0.86
**>29**	0.79	0.79	0.86	0.71	0.78	0.66	0.79	0.76	0.78	0.91

## Discussion

The results of this study illustrate the usefulness of next generation sequencing technologies for the identification of a large number of reliable SNPs. The most commonly available technologies, Illumina's Genome Analyzer and the Roche 454 FLX sequencer, were both used in this study. Other studies have previously identified a substantial number of SNPs in chickens, using traditional Sanger sequencing [25], in cattle, using Solexa sequences [13] and in pigs, using 454 sequences [23]. Our approach combined both technologies and allowed us to take advantage of the large numbers of GA sequences for an increased sequence depth, and the longer 454 reads to obtain sufficient sequence information adjacent to each SNP to be able to design oligos for the genotyping assay. Considering that approximately 30% of the pig genome had not been sequenced at the time of SNP discovery and Beadchip design, the utilization of the longer 454 reads allowed us to span this region of the genome at the same SNP density as for the 70% that was represented in genome build 7. The only caveat to this approach is the fact that because no information is available about the position of these SNPs, it was not possible to obtain a uniform distribution of SNPs in this unmapped fraction of the porcine genome. To alleviate this, the position of these SNPs within the porcine genome was predicted based on the human-porcine comparative map and the BAC-end sequences for the BACs present in the highly reliable BAC contig map [18]. Our results from mapping these SNPs to build 8 of the pig genome clearly underscore the utility of the approach. Nevertheless, these regions of the genome, in the end, will possess a less than even distribution of SNPs. The alignment of the SNPs to genome build 8 allowed us to increase the number of mapped SNPs by around 10% and we will continue to update the SNP coordinates as new porcine genome builds become available. There were 42 SNPs that mapped to a different chromosome in build 8 as compared to build 7. Inspection of the alignments for these sequences reported against the build 8 version of the genome showed that the hits identified against build 7 were still present but had been superseded by better alignments against genomic sequences introduced in build 8. This suggests that the original genomic locations identified in build 7 were actually against paralogous duplicated regions and that the greater coverage afforded by build 8 has allowed identification of the true context for these sequences.

Surprisingly, another 411 SNPs had a position assigned in build 7 but did not align to genome build 8. We presume that these “vanishing” sequences are another side-effect of the incremental BAC-by-BAC sequencing strategy being employed by the pig genome sequencing project and result from the addition of alternative duplicated regions which make the automated assembly ambiguous. As more sequence is added these regions will be resolved and the SNPs identified will be resolvable once more.

The importance of a strategy which extends the utilized reference genome is greater in species where genomic resources are less developed or for which there is no genome sequence available. In these cases, a combination of Roche 454 and Illumina GA sequencing will allow the construction of a reference genome sequence and simultaneously the detection of reliable SNPs even in the absence of a sequenced genome. Although the number of identified SNPs will be smaller because fewer reads align to a smaller reference genome, this strategy will suffice in many situations. Moreover, a recent study has also shown that a reference genome can be built and SNPs reliably identified, using only short GA sequences [Kerstens, Crooijmans, Veenendaal, Dibbits, Chin-A-Woeng, den Dunnen and Groenen, unpublished results].

The number of new porcine SNPs identified in this study exceeded 372,000, which demonstrates that the identification of large numbers of novel SNPs is now feasible in a highly efficient manner. A major limitation in genetic studies of livestock species has been the suboptimal number of available genetic markers. With the discovery of over 372,000 SNPs and the development of the PorcineSNP60 Beadchip we have alleviated this limitation, enabling whole genome association studies in pigs. Considering that the breeds used for the discovery of these SNPs include the four main breeds used in worldwide pig production (DU, PI, LR and LW) as well as the wild boar (WB), the ancestor of all modern pig breeds, it is anticipated that the PorcineSNP60 Beadchip will be highly efficient for genomic selection [14] in the pig breeding industry. The majority of the SNPs used on the PorcineSNP60 Beadchip were discovered in commercial European and US breeds and this ascertainment bias has to be taken into account when using the chip for some applications, especially when considering that domestication of pigs originated from multiple centers across Europe and Asia [26]. Hence, extensive SNP discovery in Asian pig populations could have enhanced the usefulness of the Beadchip in the Asian pig breeds. Nevertheless, the majority of the SNPs on the chip are also segregating in Chinese breeds (data not shown). However, because the extent of LD in Chinese breeds is almost an order of magnitude lower than in the commercial European/US white breeds [4], the density of SNPs on the chip will probably be too low to efficiently perform genome-wide association studies in the Chinese breeds, emphasizing the need for further SNP discovery in the pig.

The high SNP conversion rates provide an excellent indication that the majority of the SNPs identified *in silico* will translate to working SNP assays. Additionally, MAF estimates can also be precisely estimated from the GA sequence data. The frequency should be calculated on a breed by breed basis since there is large variation in the number of reads each breed contributed to the overall allele count, and the allele frequencies can vary widely between breeds. Simulations showed that given the distribution of allele frequencies, the correlations between genotype-derived and sequence-derived allele frequencies could reach values of 0.95 and higher. The maximum achieved for this study was 0.8, indicating there are additional sources of variation. One source may be the variation in constructing the pools, although previous use of pools for genotyping efforts have shown that variance introduced this way in large pools is minor (Megens, Crooijmans and Groenen, unpublished results). A second source is due to the SNPs that were identified in the sequencing, but turned out to be monomorphic in the genotyping assay. Many of these were identified with allele frequencies derived from the sequencing to be approximately 0.5 (Figure 4), strongly indicating that the reads pertain to two paralogous regions. The oligos designed for the Infinium assays may have a higher stringency than the short reads from sequencing, resulting in a locus specific assay for one of the paralogous regions that does not display population polymorphism.

One relevant question that may arise from this type of study is the decision on whether or not to use SNPs for which the minor allele is observed in a reduced number of reads. To reduce the number of false positives and maximize the conversion rate of the SNPs on the Beadchip, we adopted a conservative approach for SNP selection, and only considered SNPs for which the minor allele was represented by at least three reads. However, a significant number of additional SNPs (60,253) were identified using a very stringent threshold for the quality of the nucleotide used to call the SNP but where the minor allele was only seen twice. To detect rare SNPs with a MAF between 1–5% it will be essential to increase the depth at which each base is sequenced. However, increasing the sequence depth also directly affects the false discovery rate due to sequencing errors, further emphasizing the need to use stringent sequence quality parameters.

The overall confidence of the SNPs identified by our approach is high. (1) Application of the PorcineSNP60 Beadchip demonstrated that>95% of the predicted SNPs were validated. (2) The ratio of transitions to transversions was 2∶1 which is similar to the transition/transversion ratio observed in humans [27] and previous studies in pigs [28]–[30]. False positives detected during the SNP discovery as a result of sequencing errors should have increased the number of transversions relative to the number of transitions. (3) The error rate in the GA sequences is known to increase towards the end of the sequence reads. The observed distribution of the SNPs identified along the GA reads (Figures 1, 2) decreases towards the end of the reads indicating that we effectively filtered against false positives by increasing the stringency of removing low quality bases towards the end of the sequences. Although we did not validate any of the SNPs that were identified using a minor allele count of 2, the transition/transversion rate and the distribution of these SNPs along the GA reads indicates there should be a similar low false discovery rate in this group of SNPs. Overall, the results from the first application of the PorcineSNP60 Beadchip show it to be a valuable tool that will likely have a big impact on a variety of studies conducted in pigs.

### Conclusions

It is generally accepted that SNPs are the most common source of variation in vertebrate genomes and that significant numbers of SNPs will be needed for future studies in all species. It is now possible to conduct studies aimed at the molecular dissection of complex traits of economic importance. For this purpose, a high density porcine SNP genotyping Beadchip is now available to the community. This resource was developed using the four breeds primarily used in modern pig production and wild boar samples, which should make the Beadchip a very valuable resource for most types of pig genetic studies. The PorcineSNP60 Beadchip was tested using 158 samples and results confirm the high utility and importance that this resource will have, both in terms of working SNPs (62,621) and polymorphic SNPs (over 57K SNPs with MAF>0.05). This study also confirmed the utility of next generation sequencing technologies for the mass identification of genetic variation in any genome, including the identification of SNPs in regions of the genome that have not been previously sequenced. In addition, a very large number of additional SNPs were also identified, and even though they were not included on the Beadchip they are now publicly available for pig researchers worldwide.

## Supporting Information

Figure S1 Scatter plots for the correlation between sequence-based and genotype-based allele frequencies. The correlation plots are illustrated for the porcine breeds and RRLs analyzed in this study. The PorcineSNP60 SNPs derived from the RRLs generated were used to determine the correlations.
(9.73 MB TIF)

Table S1 The correlation plots are illustrated for the porcine breeds and RRLs analyzed in this study. The PorcineSNP60 SNPs derived from the RRLs generated were used to determine the correlations.
(0.05 MB DOC)
