# Supplementary figures and images for: Design of a High Density SNP Genotyping Assay in the Pig Using SNPs Identified and Characterized by Next Generation Sequencing Technology
Source: PLoS One. 2009 Aug 5;4(8):e6524. doi: 10.1371/journal.pone.0006524 (PMC2716536; doi:10.1371/journal.pone.0006524)

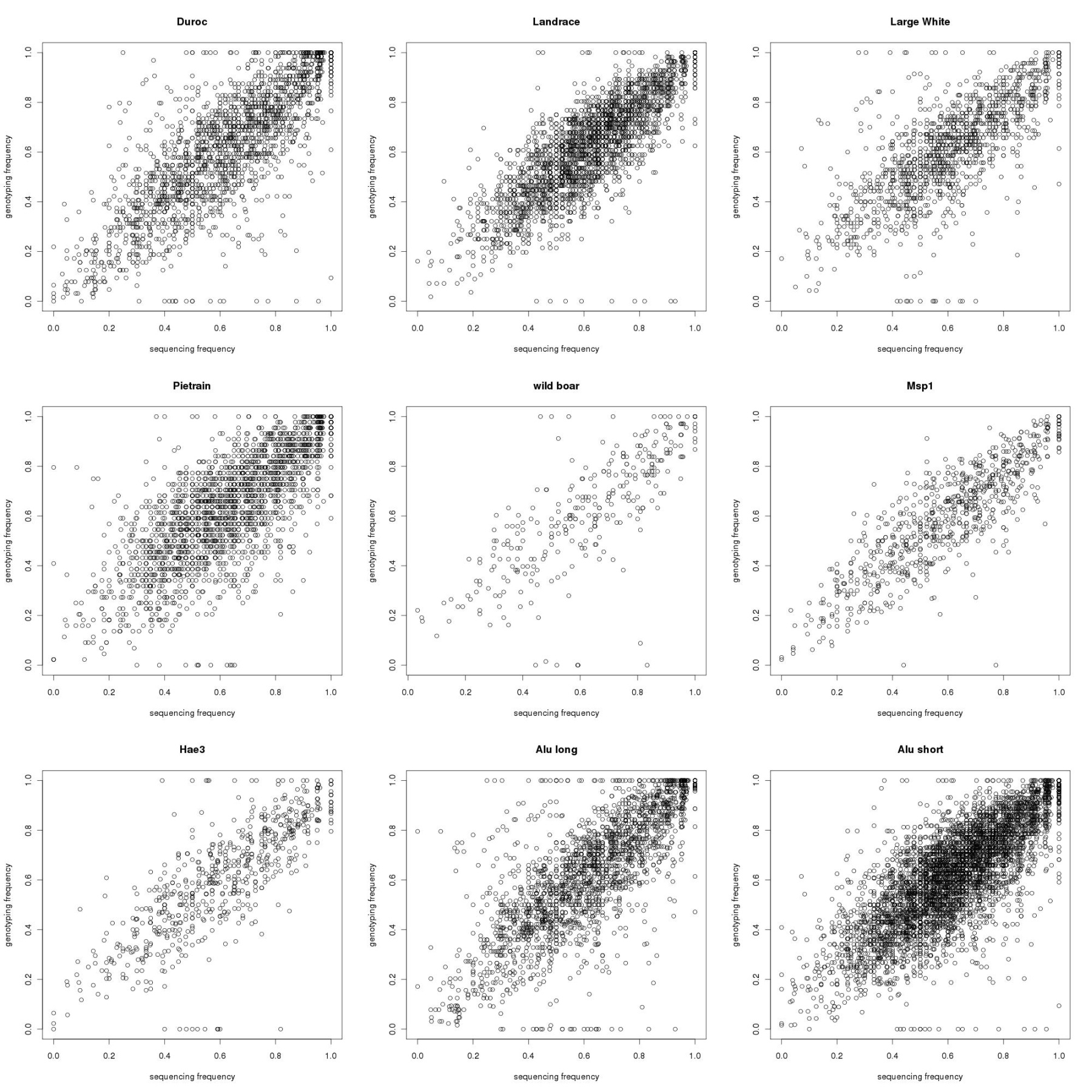

Supplement: Figure S1 — Scatter plots for the correlation between sequence-based and genotype-based allele frequencies. The correlation plots are illustrated for the porcine breeds and RRLs analyzed in this study. The PorcineSNP60 SNPs derived from the RRLs generated were used to determine the correlations. (9.73 MB TIF) [file pone.0006524.s001.tif]
