# Supplementary material for: Design of a High Density SNP Genotyping Assay in the Pig Using SNPs Identified and Characterized by Next Generation Sequencing Technology
Source: PLoS One. 2009 Aug 5;4(8):e6524. doi: 10.1371/journal.pone.0006524 (PMC2716536; doi:10.1371/journal.pone.0006524)
Supplement: Table S1 — The correlation plots are illustrated for the porcine breeds and RRLs analyzed in this study. The PorcineSNP60 SNPs derived from the RRLs generated were used to determine the correlations. (0.05 MB DOC) [file pone.0006524.s002.doc]

Supplementary Table 1 – Sources of the SNPs collected and number of SNPs from each source in the database and on the iSelect 60K+ porcine Beadchip

| **SNP source** | **Number of SNPs in database** | **Number of SNPs on Beadchip** | **SNP source description** |
| --- | --- | --- | --- |
| ALGA | 124,578 | 20,144 | *Alu*I long Genome Analyzer, Wageningen University, Netherlands |
| ASGA | 106,456 | 15,310 | *Alu*I short Genome Analyzer, Wageningen University, Netherlands |
| BGIS | 8,249 | 117 | Beijing Genomics Institute, China |
| CADI | 808 | 21 | Cambridge collection, original source is DIAS, Denmark |
| CAHM | 188 | 32 | Cambridge collection, original source is Cambridge University |
| CAIL | 259 | 17 | Cambridge collection, original source is Illumina, USA |
| CAMA | 10 | 0 | Cambridge collection, original source is animal genome database |
| CAMB | 351 | 9 | Cambridge collection, several original sources |
| CAPE | 190 | 6 | Cambridge collection, original source is PEDE database, Japan |
| CASI | 12,994 | 550 | Cambridge collection, original source is Sanger Institute, UK |
| CAUM | 51 | 0 | Cambridge collection, original source is UMB, Norway |
| CAWU | 43 | 0 | Cambridge collection, original source is Wageningen University, Netherlands |
| DBKK | 435 | 20 | dbSNP, submitted by Chungbuk National University, South Korea |
| DBMA | 262 | 21 | dbSNP, submitted by MARC |
| DBNP | 2,306 | 45 | db SNP, submitted by UAB, Spain |
| DBUN | 4,347 | 39 | dbSNP, submitted by several sources |
| DBWU | 1,077 | 96 | dbSNP, submitted by Wageningen University, Netherlands |
| DIAS | 4,826 | 1,202 | DIAS, Denmark |
| DRGA | 17,711 | 3,422 | DraI Genome Analyzer, Wageningen University, Netherlands |
| H3GA | 56,817 | 6,300 | *Hae*III Genome Analyzer, Wageningen University, Netherlands |
| INRA | 61,748 | 2,528 | Sanger sequencing SNPs, INRA, France |
| ISU | 892 | 37 | Iowa State University, USA |
| M1GA | 27,279 | 1,828 | *Msp*I Genome Analyzer, Wageningen University, Netherlands |
| MARC | 115,572 | 12,121 | 454 sequencing SNPs, MARC, USA |
| SIRI | 1,525 | 324 | Sanger Institute and Roslin Institute, UK |
| UMB | 172 | 35 | UMB, Norway |
| WUR | 136 | 8 | Wageningen University, Netherlands |
| **TOTAL** | 549,282 | 64,232 |  |
